# Neutrophil Count Predicts Malignant Cerebellar Edema and Poor Outcome in Acute Basilar Artery Occlusion Receiving Endovascular Treatment: A Nationwide Registry-Based Study

**DOI:** 10.3389/fimmu.2022.835915

**Published:** 2022-05-03

**Authors:** Chang Liu, Fengli Li, Shuai Liu, Qiong Chen, Hongfei Sang, Qingwu Yang, Kai Zhou, Wenji Zi

**Affiliations:** ^1^ Department of Neurology, Xinqiao Hospital and The Second Affiliated Hospital, Army Medical University (Third Military Medical University), Chongqing, China; ^2^ Department of Neurology, Affiliated Hangzhou First People’s Hospital, Zhejiang University School of Medicine, Hangzhou, China

**Keywords:** acute basilar artery occlusion, endovascular treatment, malignant cerebellar edema, neutrophil count, outcome

## Abstract

**Background:**

Acute basilar artery occlusion (ABAO) is known to have a poor outcome with a high rate of morbidity and mortality despite endovascular treatment (EVT), highlighting the necessities of exploring factors to limit the efficacy of EVT in these patients. Cerebellar infarctions in ABAO might progress to malignant cerebellar edema (MCE), a life-threatening complication after reperfusion, posing a secondary injury to the brainstem by mass effects. Therefore, the present research aimed to explore the impacts of MCE on a long-term outcome and investigate the prognostic factors for MCE among ABAO after EVT.

**Methods:**

In the national BASILAR registry, a total of 329 ABO patients with cerebellar infarctions treated by EVT met the inclusion criteria. The presence of MCE defined by the Jauss scale ≥4 points, was evaluated on the computed tomography performed 72 h after EVT. The adjusted odds ratio and 95% CI were obtained by logistic regression models. A favorable outcome was defined as a 90-day modified Rankin Scale score of 0–3.

**Results:**

MCE was statistically associated with the decreased incidence of a favorable outcome [adjusted odds ratio, 0.35(95% CI, 0.18-0.68), P=0.002]. The baseline National Institutes of Health Stroke Scale score, collateral circulation, neutrophil count at admission, and recanalization status were predictors for MCE and a favorable functional status at 90 days (all P<0.05). Among all inflammatory factors, the neutrophil count achieved the highest accuracy, sensitivity, and specificity for MCE. Adding the neutrophil count status into the baseline model obviously enhanced its prediction ability for MCE and favorable outcome by increasing the area under curve and achieving both net reclassification and integrated discrimination improvement (all P<0.05). Mediation analysis indicated that MCE mediated the association between the increased neutrophil count and worse functional outcome (P=0.026).

**Discussion:**

MCE acted essential roles in worsening prognosis for ABAO after EVT. A high neutrophil count at admission was linked to MCE and a poor outcome among ABAO patients, which could be further incorporated into the clinical decision-making system and guide immunomodulation therapy.

## Introduction

Acute basilar artery occlusion (ABAO) is a rare and devastating neurological disorder with high mortality that leaves a substantial part of survivors severely disabled (1). Even if recanalization by endovascular treatment (EVT) is achieved, less than 35% of patients remained functionally independent at follow-up as illustrated by the latest Basilar Artery International Cooperation Study (BASICS) and Basilar Artery Occlusion EndovascularIntervention versus Standard Medical Treatment trail (BEST) trails (2, 3). Increasing focus was required to promote an understanding for the reasons of poor outcome and factors that limit the efficacy of EVT.

Brainstem impairment after the occluding basilar artery was regarded as the major contributor for a poor outcome; nevertheless, the phenomena of cerebellar infarctions were frequently observed in ABAO patients that might progress to malignant cerebellar edema (MCE), which could result in obstructive hydrocephalus and pose a secondary injury to the brainstem by mass effects (4). Previous studies have reported that the mortality rate of MCE was more than three times higher than that of non-MCE among pure acute cerebellar infarction patients (5, 6). However, these related previous study focused on anterior circulation stroke, while the basilar artery supplied blood to the infratenttorial structure , ABAO was excluded in these literatures on MCE, and thus, the evidence regarding the relationship between MCE and the outcome and predictive factors for the MCE in ABAO after EVT were scarce (5, 6).

According to findings from animal studies and cross-sectional observation studies, inflammatory cells could act as critically pathogenic but intervenable factors for brain tissue edema, compared to other unchangeable predictors such as the collateral circulation status and stroke severity degrees (7, 8). However, the patients recruited in previous literatures that investigated the associations of inflammation with brain edema, were anterior circulation infarction, while the basilar artery supplied blood to the infratentorial structure (7–9). The obvious histological and anatomical differences of the cerebellum from the cerebrum, including cellular components, feeding arteries, and vessel autoregulation methods, might imply the distinct roles of inflammatory factors in promoting cerebellar edema from cerebral tissue edema (10). The limited subtentorial residual space also suggested the decreased tolerance ability for mass effects among ABAO patients than an anterior circulation stroke. Uncertainties remained about the relationship between inflammation markers and MCE in ABAO after EVT.

Therefore, based on our preceding national registry of BASILAR (11), we ought to evaluate the impacts of MCE on the clinical outcome in ABAO patients after EVT and explore the predictors for MCE, especially the roles of inflammation factors in determining MCE and the functional outcome among these individuals.

## Material and Methods

### Study Design and Participants

The BASILAR study was a nationwide, prospective registry of EVT plus medical management vs. medical management alone for patients confirmed with an acute symptomatic and radiological ABAO from 47 comprehensive stroke centers in China between January 2014 and May 2019. The details of the study protocol have been previously published (11). We included the ABAO subjects 1) with confirmed cerebellar infarction based on magnetic resonance imaging (MRI) or computed tomography (CT) perfusion at admission, or the corresponding follow-up imaging scanning (5), and 2) treated by EVT. To reduce the influence of drugs and related medication disease history on inflammatory cells, the main exclusion criteria were 1) taking antibiotics, 2) with an infection within 7 days before admission, 3) with major trauma or surgery events within 28 days before admission, 4) with chronic inflammatory diseases, and 5) accepting corticosteroid treatment. From 829 patients in the BASILAR registry, a total of 329 patients were enrolled in the present study.

### Standard Protocol Approvals, Registrations, and Patient Consent

The BASILAR was registered on the Chinese Clinical Trial Registry (ChiCTR1800014759). The study was approved by the research board at each participating center, and informed consent was obtained from all patients or their authorized representatives.

### Procedures and Data Collection

We retrospectively collected data on baseline characteristics, including age, sex, vascular risk factors (diabetes mellitus, hypertension, atrial fibrillation, and dyslipidemia), National Institutes of Health Stroke Scale (NIHSS) at admission, and posterior circulation-Alberta Stroke Program Early scores (PC-ASEPCTS). The collateral circulation status was assessed by the posterior circulation collateral score (PC-CS) based on the presence of potential collateral pathways on CT angiography (12). Successful reperfusion was defined as a modified thrombolysis-in-cerebral-infarction (mTICI) score higher than 2a at the end of the intervention. Malignant cerebellar edema (MCE) was quantified by the score of Jauss higher than 3 (≥4) from non-contrast CT within 72 h after EVT (13). Each imaging scan for Jauss score was separately interpreted by 2 trained neuroradiologists (KZ and SL). In the cases of discrepancies, a third neuroradiologist (KZ) confirmed the MCE status. All neuroradiologists were blinded to clinical outcomes when performing imaging data analysis.

Whole blood samples were collected from patients at admission. Five key potential blood-based biomarkers including the neutrophil count, platelet count, lymphocyte count, and their combination ratios [neutrophil-to-lymphocyte ratio (NLR) and platelet-to-lymphocyte ratio (PLR)] were obtained. Patients were categorized into high or low neutrophil count groups based on the median value of the neutrophil count in enrolled patients.

The primary functional outcomes at follow-up were assessed using the 90-day modified Rankin Scale (mRS). A score on the 90-day mRS of 0-3 was defined as a favorable outcome. Intracerebral hemorrhages were evaluated according to the Heidelberg Bleeding Classification (14). Symptomatic intracranial hemorrhage (SICH) was defined as a newly observed intracranial hemorrhage, leading to an increase of 4 points on the NIHSS before worsening or an increase of 2 points in one category (2, 11).

### Statistical Analysis

The proportion tests for categorical variables were performed using the chi-square test. Depending on the normality of the distribution as assessed by the Kolmogorov–Smirnov test, continuous variables were compared using Student’s t-test for independent samples, or the Mann–Whitney U test or Kruskal–Wallis test for non-normal data. Data are presented as mean ± standard deviation, median [interquartile range(IQR)], or a number (percentage), where appropriate. The interobserver agreement for MCE status assignment was evaluated using the weighted κ statistic.

To determine the independent prognostic factors for favorable outcomes, binary and multivariable logistic regression analyses were performed and these results were summarized as odds ratios (ORs) with 95% confidence intervals (CIs). An ordinal regression model was performed to calculate the common OR for a shift toward a 1-point improvement of mRS. We also analyzed the heterogeneity of the effects of the neutrophil count status within subgroups based on the sex, age (≤60 or >60 years), mTICI (0–2a or 2b–3), NIHSS score (<25 or ≥25), and PC-CS (≤6 or >6). The area under the receiver operating characteristic (ROC) curves (AUCs) were calculated and compared using DeLong’s test. The incremental effects of the neutrophil count status for outcome prediction were examined using the net reclassification improvement (NRI) algorithm and the integrated discrimination improvement (IDI) algorithm, with the baseline model as a reference. We performed a sensitivity analysis to explore the impacts of the neutrophil count on the outcomes in cohorts without any hemorrhage, sICH, and poor reperfusion level (mTICI ≤ 2a). The predictive roles of neutrophils for the long-term outcome were also explored in all ABAO patients after EVT in the BASILAR registry based on logistics analysis. A 2-tailed P<0.05 was considered statistically significant. All statistical analyses were performed using the R software version 4.0.2 (https://www.r-project.org).

## Results

### Baseline Characteristics

The baseline clinical manifestations of enrolled patients were shown in **Supplemental Table 1**. The average age was 63.86 years, and 78.12% of them were men. Among enrolled patients, a total of 89 (27.05%) achieved favorable long-term outcomes (90-day mRS ≤ 3).

With a weighted κ value of 0.77 (95% CI, 0.73–0.80), the interobserver agreement for the grading of MCE status on follow-up non-contrast computed tomography (NCCT) images was good. Patients were stratified into two groups according to their MCE level (**Table 1** and **Supplemental Figure 1**). The proportion of MCE among ABAO patients with cerebellar infarctions treated by EVT was 36.17% (N=119). Significant differences in the proportions of satisfied mTICI level, PC-CS score, onset to recanalization time, and neutrophil count were detected between these MCE and non-MCE groups (all P<0.05).

**Table 1:** Clinical features for enrolled patients stratified by different MCE levels.

	All patients (N=329)	Non-MCE (N=210)	MCE (N=119)	P
Age, years, mean ± SD	63.86 ± 10.83	64.21 ± 11.24	63.24 ± 10.09	0.436
Men, (n%)	257 (78.12)	158 (75.24)	99 (83.19)	0.094
Baseline NIHSS, median (IQR)	27.00 (17.00,33.00)	24.00 (14.00,32.00)	30.00 (23.00,34.00)	<0.001
Initial PC-ASPECTS, median (IQR)	8.00 (6.00, 9.00)	8.00 (7.00, 9.00)	7.00 (6.00, 9.00)	0.017
Admission SBP, mmHg, mean ± SD	150.97 ± 25.79	149.55 ± 24.84	153.46 ± 27.31	0.187
Admission DBP, mmHg, mean ± SD	86.74 ± 15.67	86.43 ± 14.83	87.28 ± 17.11	0.638
24 h NIHSS after EVT, median (IQR)	29.00 (15.00, 35.00)	21.50 (11.00, 32.00)	34.00 (28.00, 36.00)	<0.001
7 d NIHSS after EVT, median (IQR)	24.00 (9.00, 36.00)	16.00 (6.00, 31.00)	35.00 (28.00, 36.00)	<0.001
Intravenous thrombolysis, (n%)	71 (21.58)	46 (21.90)	25 (21.01)	0.849
Pre-onset mRS, (n%)				
0	284 (86.32)	178 (84.76)	106 (89.08)	0.448
1	31 (9.42)	23 (10.95)	8 (6.72)	
2	14 (4.26)	9 (4.29)	5 (4.20)	
Decompressive craniectomy, (n%)	11 (3.34)	0 (0.00)	11 (9.24)	<0.001
History of risk factors, n (%)				
Hypertension	231 (70.21)	146 (69.52)	85 (71.43)	0.717
Diabetes mellitus	75 (22.80)	50 (23.81)	25 (21.01)	0.516
Dyslipidemia	109 (33.13)	68 (32.38)	41 (34.45)	0.701
Atrial fibrillation	59 (17.93)	39 (18.57)	20 (16.81)	0.688
TIA	3 (0.91)	1 (0.48)	2 (1.68)	0.297
Laboratory results, median (IQR)				
Neutrophil, 10^9^/L	9.87 (7.28, 12.40)	9.15 (6.60, 11.29]	11.85 (8.88,14.26)	<0.001
Lymphocyte, 10^9^/L	1.09 (0.80, 1.60)	1.10 (0.80, 1.61)	1.06 (0.85,1.56)	0.826
NLR	8.83 (5.35, 13.66)	7.80 (4.64, 12.88)	10.75 (6.62,15.24)	0.001
Platelet count, 10^9^/L	216.00 (177.00,250.00)	213.00 (178.00, 245.00)	221.00 (174.50,266.25)	0.109
PLR	180.83 (131.09, 277.61)	178.46 (127.93, 275.63)	186.92 (137.68, 284.66)	0.272
TOAST classification, n (%)				0.392
LAA	227 (69.00)	138 (65.71)	89 (74.79)	
CE	73 (22.19)	52 (24.76)	21 (17.65)	
SOE	7 (2.13)	5 (2.38)	2 (1.68)	
SUE	22 (6.69)	15 (7.14)	7 (5.88)	
Imaging parameters				
Occlusion site, n (%)				0.105
BA distal	96 (29.18)	68 (32.38)	28 (23.53)	
BA middle	108 (32.83)	70 (33.33)	38 (31.93)	
BA proximal	56 (17.02)	36 (17.14)	20 (16.81)	
V4	69 (20.97)	36 (17.14)	33 (27.73)	
PC-CS score, median (IQR)	4.00 (3.00, 6.00)	5.00 (3.25, 6.00)	4.00 (2.00, 5.00)	<0.001
Reperfusion status, n (%)				
mTICI				<0.001
0-2a	60 (18.24)	29 (13.81)	31 (26.05)	
2b-3^a^	269 (81.76)	181 (86.19)	88 (73.95)	
Treatment delay, min, median (IQR)				
Onset to puncture	334.00 (219.75, 497.00)	315.00 (203.00, 475.00)	368.00 (262.50, 557.00)	0.004
Puncture to recanalization	107.00 (72.00, 152.25)	103.00 (69.00, 144.00)	112.00 (86.00, 164.00)	0.032
Onset to recanalization	456.00 (327.75, 633.00)	435.00 (302.75, 585.00)	504.00 (362.00, 725.50)	0.001

a mTICI score of 2b or 3 indicates complete recanalization.

PC-CS, posterior circulation collateral system score; BA, basilar artery; mTICI, modified thrombolysis in cerebral infarction; PCA, posterior cerebral artery; V4, V4 segment of vertebral artery; CE, cardioembolism; NIHSS, National Institutes of Health Stroke Scale; PC-ASPECTS, posterior circulation Alberta Stroke Program Early CT Score; SBP, systolic blood pressure; DBP, diastolic blood pressure; SOE, stroke of other determined cause; SUE, stroke of undetermined cause; TIA, transient ischemic attack; TOAST, Trial of ORG 10172 in Acute Stroke Treatment.

### The Impacts of Malignant Cerebellar Edema on Clinical Outcome for Acute Basilar Artery Occlusion With Cerebellar Infarctions

As shown in **Table 2**, favorable outcomes were least likely among patients with MCE, with much lower percentages of 90-day mRS 0-3 and a decreased median value of 90 day-mRS than the non-MCE group (non-MCE vs. MCE: 90-day mRS ≤ 3, 36.67% vs. 10.08%, P<0.001; mRS, 5(2-6) vs. 6(5-6), P<0.001). In both the unadjusted model and multivariate analysis with adjustment for confounders, MCE was found to be significantly associated with the 90-day mRS value [adjusted common OR, 2.86[95% CI, 1.64–4.92), P<0.001], long-term favorable outcome [adjusted OR, 0.35(95% CI, 0.18–0.68), P=0.002], and mortality at 90 days [adjusted OR, 3.24(95% CI, 1.79–5.95), P<0.001].

**Table 2:** The impacts of MCE on clinical outcome.

	MCE	No./No. (%)	P-value	OR (95%CI)	P	Adjusted OR (95%CI)	P
90-day mRS 0-3, n (%)	Non-MCE	77 (36.67)	<0.001^c^	0.19 (0.10-0.36)	<0.001	0.35 (0.18,0.68)^g^	0.002
	MCE	12 (10.08)					
mRS, median (IQR)	Non-MCE	5 (2-6)	<0.001^d^	5.37 (3.35,8.82)^e^	<0.001	2.86 (1.69,4.92)^h^	<0.001
	MCE	6 (5-6)					
Mortality in hospital, n (%)	Non-MCE	34 (16.19)	0.005^c^	2.16 (1.26,3.71)	0.005	1.19 (0.63,2.24)^g^	0.588
	MCE	35 (29.41)					
Mortality at 90 d, n (%)	Non-MCE	72 (34.29)	<0.001^c^	5.69 (3.47,9.51)	<0.001	3.24 (1.79,5.95)^g^	<0.001
	MCE	89 (74.79)					
ΔNIHSS at 24 h^a^, median (IQR)	Non-MCE	-5.00 (-11.00, 2.00)	<0.001^d^	4.26 (2.17,6.35)^f^	<0.001	3.38 (1.31,5.44)^i^	0.001
	MCE	2.00 (0.00, 6.00)					
ΔNIHSS at 5-7 d^b^, median (IQR)	Non-MCE	0.00 (-5.00 to 2.00)	<0.001^d^	7.29 (4.72,9.86)^f^	<0.001	5.96 (3.46,8.46)^i^	<0.001
	MCE	2.00 (0.00 to 6.00)					

a Change from NIHSS at admission from NIHSS at 24 h after EVT.

b Change from NIHSS at admission from NIHSS at 5-7 days after EVT.

c Chi-square test.

d Wilcoxon test.

e Common OR.

f ß-values were estimated from a univariate linear regression model.

g Adjusted OR; adjusted estimates of outcome were calculated using multiple regression, taking the following variables into account: baseline NIHSS score, baseline PC-ASPECTS, neutrophil count, mTICI, PC-CS score, occlusion sites, and onset to recanalization time.

h Adjusted common OR; adjusted estimates of outcome were calculated using multiple regression, taking the following variables into account: baseline NIHSS score, baseline PC-ASPECTS, neutrophil count, mTICI, PC-CS score, occlusion sites, and onset to recanalization time.

i ß-values were estimated from a multivariable linear regression model; adjusted estimates of outcome were calculated using multiple regression, taking the following variables into account: baseline NIHSS score, baseline PC-ASPECTS, neutrophil count, mTICI, PC-CS score, occlusion sites, and onset to recanalization time.

NIHSS, National Institutes of Health Stroke Scale; mRS, modified Rankin Scale score at 90 days; MCE, malignant cerebellar edema; PC-CS, posterior circulation collateral system score; mTICI, modified thrombolysis in cerebral infarction; PC-ASPECTS, posterior circulation Alberta Stroke Program Early CT Score.

### Predictors for Malignant Cerebellar Edema and Clinical Outcome

As shown in **Table 3**, univariate logistic regression showed that the neutrophil count, NIHSS score at baseline, PC-ASPECTS, PC-CS, mTICI and onset to recanalization time were related with the incidences of MCE (all P<0.05). Multivariable logistic regression that included predictors identified by the univariate analysis (at P<0.05) found the following independent predictors of MCE: NIHSS at admission [adjusted OR, 1.04(5% CI, 1.01-1.07), P = 0.013], neutrophil count [adjusted OR 1.1(95% CI, 1.09-1.25), P < 0.001], mTICI [adjusted OR, 0.80(95% CI, 0.67-0.95), P<0.001], and PC-CS score [adjusted OR, 0.77(95% CI, 0.66-0.88), P<0.001]. Similarly, our results detected that the neutrophil count [adjusted OR, 0.83(95% CI, 0.76-0.91), P<0.001], NIHSS [adjusted OR, 0.83(95% CI, 0.76-0.91), P<0.001], and mTICI [adjusted OR, 1.74(95% CI, 1.31-2.37), P<0.001] were still associated with a favorable long-term outcome (90-day mRS ≤ 3).

**Table 3:** Predictors for MCE and favorable outcome (mRS ≤ 3) in enrolled ABAO patients with cerebellar infarctions treated by EVT.

	MCE				Favorable outcome (mRS ≤ 3)				
	Univariate analysis		Multivariate analysis		Univariate analysis			Multivariate analysis	
	OR (95%CI)	p-value	Adjusted OR (95%CI)	p-value	OR (95%CI)	p-value	Adjusted OR (95%CI)		p-value
Age	0.99 (0.97-1.01)	0.435			0.99 (0.97-1.02)	0.515			
Sex	1.63 (0.93-2.94)	0.095			0.62 (0.36-1.10)	0.099			
Dyslipidemia	1.10 (0.68-1.76)	0.701			1.27 (0.76-2.11)	0.355			
Diabetes mellitus	0.85 (0.49-1.45)	0.561			0.61 (0.32-1.11)	0.120			
Hypertension	1.10 (0.67-1.81)	0.717			1.21 (0.71-2.10)	0.496			
Atrial fibrillation	0.89 (0.48-1.59)	0.689			1.23 (0.65-2.55)	0.510			
Lymphocyte	0.90 (0.64-1.23)	0.510			1.29 (0.92-1.80)	0.130			
Neutrophil count	1.18 (1.11-1.27)	<0.001	1.17 (1.09-1.25)	<0.001	0.80 (0.73-0.86)	<0.001	0.83 (0.76-0.91)		<0.001
NLR	1.01 (1.00-1.04)	0.10			0.94 (0.89-0.97)	0.003			
SBP	1.01 (0.99-1.01)	0.187			1.00 (0.99-1.01)	0.467			
DBP	1.00 (1.00-1.04)	0.637			0.99 (0.98-1.01)	0.298			
TOAST									
LAA	Reference	NA			Reference	NA			
CE	0.63 (0.35,1.10)	0.109			1.59 (0.89,2.80)	0.111			
SOE	0.62 (0.09,2.95)	0.573			0.51 (0.03,3.07)	0.536			
SUE	0.72 (0.27,1.79)	0.498			1.43 (0.52,3.56)	0.463			
Occlusion Sites									
BA distal	Reference	NA			Reference	NA	Reference		NA
BA middle	1.32 (0.73,2.39)	0.360			0.44 (0.24,0.80)	0.008	0.28 (0.12, 0.63)		0.003
BA proximal	1.35 (0.66,2.72)	0.403			0.44 (0.20,0.91)	0.031	0.48 (0.17,1.25)		0.140
V4	2.23 (1.17,4.28)	0.015			0.31 (0.14,0.63)	0.002	0.29 (0.11,0.74)		0.011
Thrombolysis treatment	0.98 (0.70-1.34)	0.883			1.05 (0.74-1.48)	0.762			
Preonset mRS	0.82 (0.49-1.32)	0.428			0.69 (0.36-1.18)	0.201			
Onset to recanalization time	1.00 (1.00-1.01)	0.003	1.00 (1.00-1.00)	0.005	1.00 (1.00-1.00)	0.184			
NIHSS baseline	1.06 (1.03-1.09)	<0.001	1.04 (1.01-1.07)	0.013	0.91 (0.88,0.93)	<0.001	0.92 (0.89-0.95)		<0.001
PC-ASPECTS	0.83 (0.72-0.96)	0.011			1.70 (1.42,2.07)	<0.001	1.62 (1.29-2.06)		<0.001
mTICI	0.76 (0.65-0.88)	<0.001	0.80 (0.67-0.95)	0.011	1.83 (1.46,2.35)	<0.001	1.74 (1.31-2.37)		<0.001
PC-CS score	0.74 (0.65-0.84)	<0.001	0.77 (0.66-0.88)	<0.001	1.32 (1.16,1.5)	<0.001	1.22 (1.01,1.47)		0.050

PC-CS, posterior circulation collateral system score; BA, basilar artery; mTICI, modified thrombolysis in cerebral infarction; PCA, posterior cerebral artery; V4, V4 segment of vertebral artery; CE, cardioembolism; NIHSS, National Institutes of Health Stroke Scale; PC-ASPECTS, posterior circulation Alberta Stroke Program Early CT Score; SBP, systolic blood pressure; DBP, diastolic blood pressure; SOE, stroke of other determined cause; SUE, stroke of undetermined cause; TIA, transient ischemic attack; TOAST, Trial of ORG 10172 in Acute Stroke Treatment, NA, Not available.

### Association of Neutrophil Count With Malignant Cerebellar Edema and Long-Term Outcome

**Figure 1:**
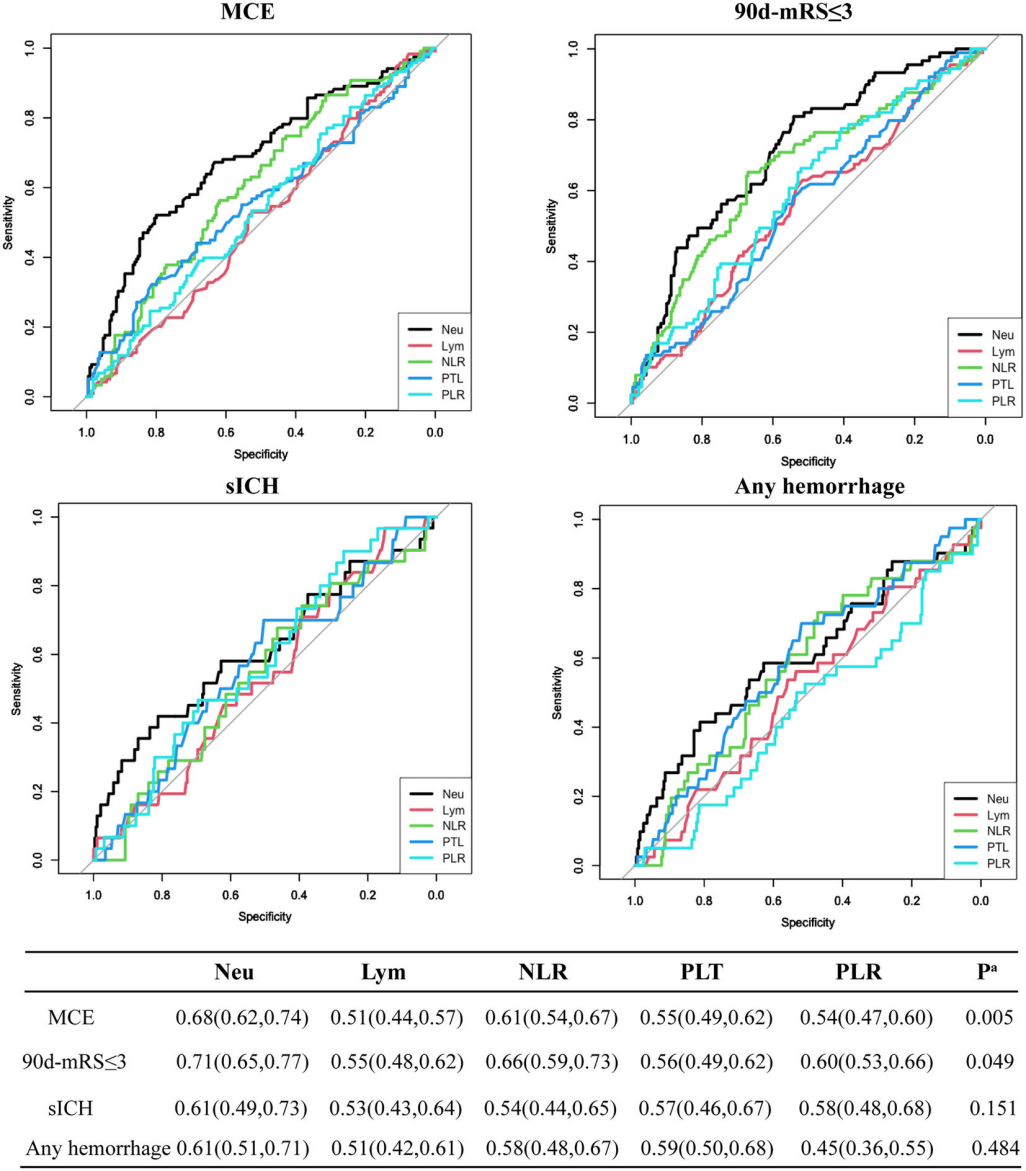
Predictive values of neutrophil count (Neu), leukocyte count (Lym), platelet count (PTL), PLR, and NLR for 4 endpoints including MCE, long-term favorable outcome, sICH, and any hemorrhage.

With the development of immunotherapy, inflammation cells might act as novel potential therapeutic targets for stroke at admission, compared to other unchangeable predictors for MCE identified above, such as NIHSS, the circulation level, and mTICI status (7, 8, 15). Thus, we compared the predictive values of inflammation factors for 4 endpoints and found that the neutrophil count achieved the highest AUC value when predicting MCE and 90-day mRS ≤3 [MCE: neutrophils vs. NLR, 0.68(0.62, 0.74) vs. 0.61(0.54,0.67), P=0.005 by using DeLong’s test; favorable outcome: neutrophils vs. NLR, 0.71(0.65, 0.77) vs. 0.66(0.59, 0.73), P=0.049 by using the DeLong’s test; **Figure 1**].

**Figure 2:**
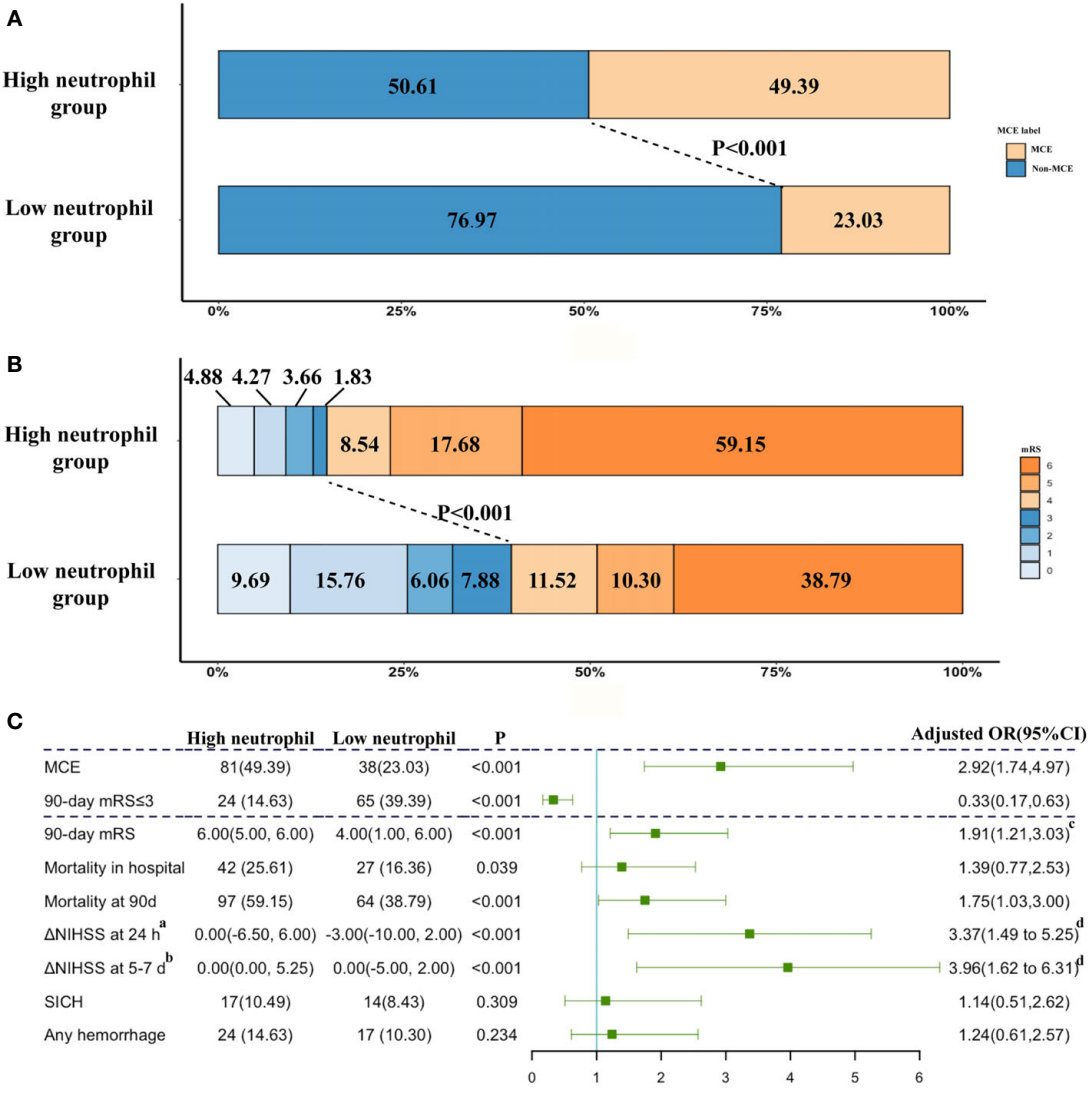
The impacts of neutrophil count on clinical outcome. **(A)** Distribution of the MCE across patients with different neutrophil count status; **(B)** Distribution of the mRS scores at 90 days according to the trichotomized neutrophil count status. **(C)** Adjusted logistic regression models evaluated the associations between neutrophil level with outcomes. ^a^ Change from NIHSS at admission from NIHSS at 24 h after EVT. ^b^ Change from NIHSS at admission from NIHSS at 5-7 days after EVT. ^c^Adjusted common OR; adjusted estimates of outcome were calculated using multiple regression, taking the following variables into account: baseline NIHSS score, baseline PC-ASPECTS, neutrophil count, mTICI, PC-CS score, occlusion sites, and onset to recanalization time. ^d^ß-values were estimated from a multivariable linear regression model; adjusted estimates of outcome were calculated using multiple regression, taking the following variables into account: baseline NIHSS score, baseline PC-ASPECTS, neutrophil count, mTICI, PC-CS score, occlusion sites, and onset to recanalization time. NIHSS, National Institutes of Health Stroke Scale; mRS, modified Rankin Scale score at 90 days.

These ABAO patients with cerebellar infarctions after EVT were further divided into the high neutrophil group and low neutrophil group by the median value of neutrophils (9.87×10^9^/L) in enrolled patients (**Supplemental Table 2**). The ratio of MCE significantly increased, while the incidence of favorable outcome obviously reduced in patients with a high neutrophil status (high neutrophil group vs. low neutrophil group: MCE: 49.39% vs. 23.03%, **Figure 2A**; favorable outcome: 14.63% vs. 39.39%, **Figure 2B**). As shown in **Figure 2C**, based on multivariable analysis, the high neutrophil level was remarkably related with 90-day mRS ≤ 3 [adjusted OR, 0.33(95% CI, 0.17-0.63)] and MCE [adjusted OR, 2.92(95% CI, 1.74-4.97)].

**Figure 3:**
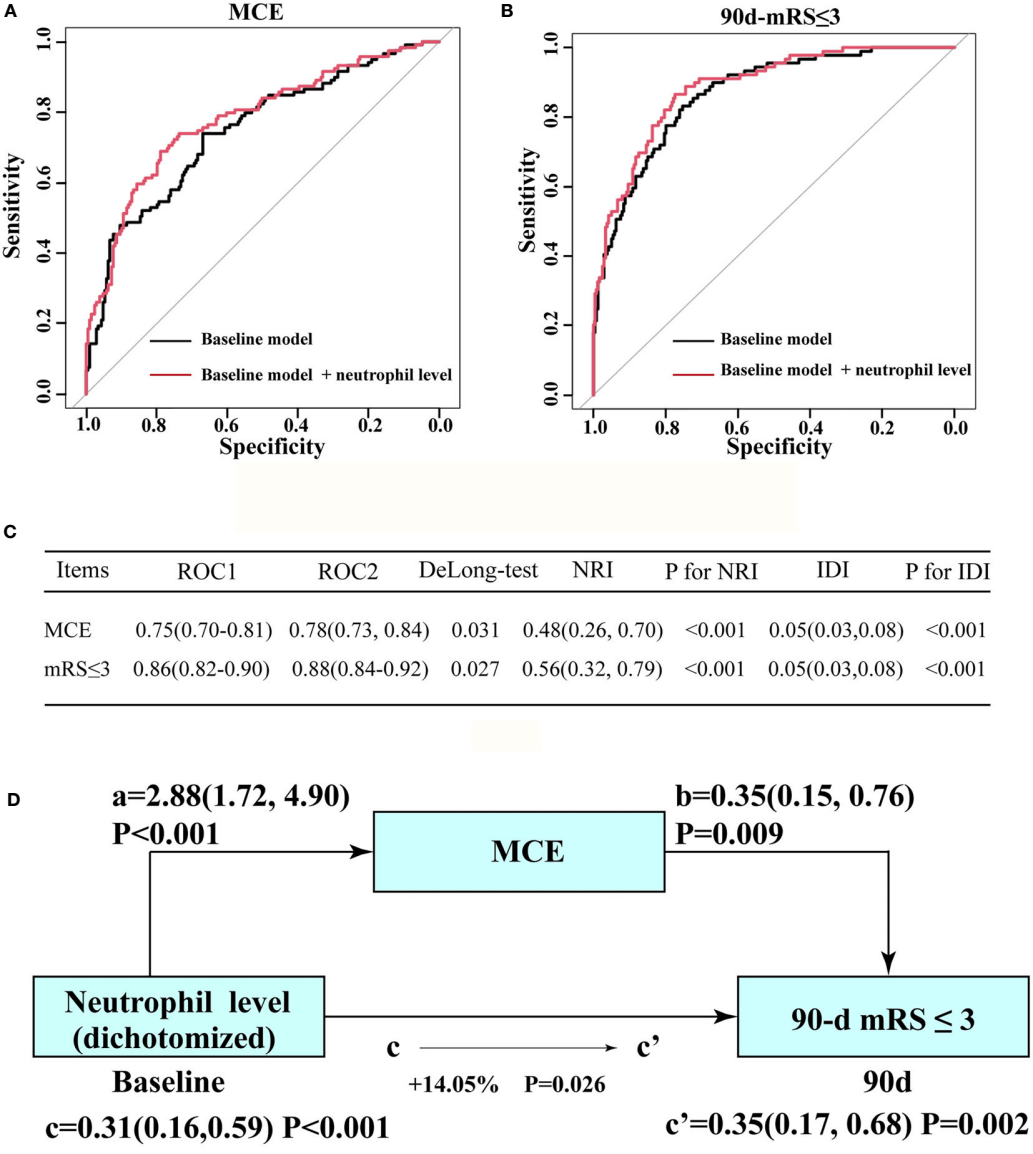
Incremental effect of the neutrophil count level on the predictive value of the baseline model for favorable outcome and MCE. **(A)** ROC curves for baseline model and novel model count level when predicting long-term outcome. **(B)** ROC curves for baseline model (ROC1) and novel model (ROC2: adding neutrophil count level into the baseline model) when predicting MCE. **(C)** Results from DeLong’s test, the NRI, and IDI by comparing ROC curves in **(A, B)**, separately. **(D)** Mediation analysis by MCE of the associations between neutrophil count level and clinical outcome. a, regression coefficient of the association between neutrophil count and MCE; b, regression coefficient of the association between MCE and clinical outcome; c, regression coefficient of the association between neutrophil count level and clinical outcome; c’, regression coefficient of the association between neutrophil count level and clinical outcome, using neutrophil count level and MCE as additional independent variables. The percentage difference of the coefficients (1-c/c’) is shown. a, b, c, and c’ were evaluated by adjusted OR, taking the following variables into account: baseline NIHSS score, baseline PC-ASPECTS, mTICI, PC-CS score, occlusion sites, and onset to recanalization time.

The addition of the neutrophil status significantly increased the prediction ability of the baseline model for MCE in enrolled individuals and yielded a statistically elevated AUC value [baseline model vs. baseline model + high neutrophil status (dichotomized): 0.75(95% CI: 0.70–0.81) vs. 0.78(95% CI: 0.73–0.84), P=0.031 by using DeLong’s test; **Figure 3A**]. Significant improvements in risk reclassification and discrimination were also detected after adding the neutrophil status (dichotomized) into the baseline model, with an NRI of 0.48[95% CI: 0.26–0.70] and an IDI of 0.05 [95% CI: 0.03–0.08] (**Figure 3C**). The improved roles of neutrophils for the baseline model in predicting a long-term outcome was also observed [baseline model vs. baseline model + high neutrophil status (dichotomized): 0.86(95% CI: 0.82–0.90) vs. 0.88(95% CI: 0.84–0.92), P=0.027 by using the DeLong’s test; **Figure 3B**]. After including MCE as a mediator, we observed a significant partial mediation effect for MCE on higher neutrophil count (dichotomized)-related effects on the 90-day functional outcome in enrolled patients (estimated proportion mediated by MCE: 14.05%, P=0.026, **Figure 3D**).

### Subgroup and Sensitive Analysis

None of the interactions by variables including age, sex, NIHSS, mTICI, PC-ASPECTS, and PC-CS score between the neutrophil count level and MCE and the long-term outcome were statistically significant (**Supplemental Figures 2A, B**). Sensitivity analysis showed no modifications of the effects of neutrophils on outcomes after the exclusion of symptomatic hemorrhagic, transformation, and unsuccessful revascularization. The details of the analysis are shown in **Supplemental Table 3**. Given that the proportion of men was significantly higher than that of women, we further explored the prognostic value of neutrophils in different genders in **Supplemental Figure 3**. The high neutrophil count was positively related with MCE and poor outcome in both female and male patients. Neutrophil counts were not significantly altered between female MCE patients and male MCE patients and between female poor outcome patients and male poor outcome patients.

Among all ABAO patients after EVT in the BASILAR registry, the impacts of the neutrophil status on outcomes were also evaluated. As shown in **Supplemental Table 4**, the frequency of a favorable outcome was obviously decreased in the high neutrophil group [low neutrophil group vs. high neutrophil group: 133(40.06) vs. 56(22.05), P <0.001]. Based on multivariate analysis, the high neutrophil group was still an independent predictor for a 90-day favorable outcome [adjusted OR with 95% CI: 0.49 (0.30-0.78), P=0.002] in all ABAO patients. To further investigate the clinical value of findings in the present study, we classified enrolled patients into the normal range group (2-8 × 10(9)/L), elevated group (8-9.87 × 10(9)/L), and extremely high group (≥9.87 × 10(9)/L) in **Supplemental Tables 5, 6**. No patients enrolled in the present research had a neutrophil count below 2 × 10(9)/L. In **Supplemental Table 5**, we compared the baseline characteristics among the three groups. As shown in **Supplemental Table 6**, we found that the elevated neutrophil count group had similar effects on clinical outcomes compared with the normal range group (all P>0.05), while the extremely high neutrophil counts significantly reduced the proportion of favorable prognosis [aOR 0.37, 95%CI (0.18, 0.75), P=0.006] and increased the ratio of MCE [aOR 2.36, 95%CI (1.29, 4.40), P=0.006].

## Discussion

Based on a multi-centered cohort derived from the BASILAR registry, this is, to our knowledge, the first study to explore the associations between MCE and clinical outcomes, and investigate predictors for MCE in ABAO patients with cerebellar infarctions treated by EVT. Our results revealed that 1) the presence of MCE was associated with 0.35-folds for favorable outcome and 3.24-folds for mortality among these patients; 2) the neutrophil count was a more reliable predictor of MCE compared with other inflammatory markers; 3) the prognostic roles of a high neutrophil status for a poor long-term outcome could be partially explained by its roles in promoting MCE; 4) adding a neutrophil level to a conventional model obviously improved its prediction ability for MCE and outcome in enrolled ABAO patients.

The incidence of MCE was estimated to range from 4% to 39% among all cases of pure cerebellar infarctions, while the MCE in ABAO with cerebellar infarctions were less intensively explored, who were usually excluded in previous literatures (5, 6, 16). In our study, the rate of MCE in ABAO with cerebellar infarctions was 36.17%, which was comparable to the previously reported incidence of MCE in pure cerebellar infarctions (5, 16). However, the rates for poor outcome and mortality in ABAO individuals with MCE were 89.92% and 74.79%, respectively, which were much higher than ABAO without MCE and pure cerebellar infarctions with MCE (16). Aside from the concomitant brain stem injury after occluding basilar artery, the relative low incidence of decompression craniectomy (N=11) might also be the important reason for the worse prognosis of ABAO with MCE in present research (4, 5). In addition, compared with previous literatures focusing on brain stem infarcts and illustrating that brainstem damage degree determined the outcomes for these populations, our results provided a new evidence of involving the cerebellum as a reliable prognosticator of poor outcome due to the posterior fossa mass effects of MCE (17, 18). Patients with cerebellar infarcts may require prompt detection during pretreatment imaging, closely monitoring for the early detection of MCE, and even considerations for pre-emptive neurosurgery (6).

As a marker of systemic inflammation, the neutrophil count is easily obtained from routine laboratory data without an additional technique or cost. In line with findings in previous researches on the anterior circulation stroke, our research found that an elevated neutrophil count was a strong prognostic factor for worse clinical outcomes in ABAO patients after EVT (19, 20). Physically, the neutrophils in infarcted regions would obviously block the microvascular network, reduce the brain blood flow of microvessels, and aggravate tissue damage (21). Both *in vitro* and *in vivo* experiments also demonstrated that the myeloperoxidase and neutrophil extracellular traps from neutrophils could directly increase neuronal death and lead to augmented final infarction volumes (22). The elaborated functions of neutrophils in ischemic lesions have led neutrophils to be promising therapeutic targets to reduce damage after stroke (23).

Mediation analysis in our study suggested that MCE might be an important mechanism linking peripheral inflammation to a poor outcome for ABAO patients after EVT. The blood–brain barrier (BBB) was disrupted early after stroke, which would facilitate the infiltration of peripheral immune cells into the infarct regions to further potentiate damages for constituents in BBB. Among these immune cells, the neutrophil count in infarction tissues displayed an exponential increase within a few hours after the symptom onset and remained elevated for 1 week, while lymphocytes extravasated into these regions in a smaller count and reached maximum accumulation several days later (24). Thus, due to the fact that brain tissue edema most likely occurred within 3 days after EVT according to previous reports (5, 16), the fast accumulation of neutrophils might play critical roles in promoting injury. In contrast with findings from the Daniela group, which demonstrated that neutrophils and the neutrophil-to-lymphocyte ratio (NLR) showed identical predictive performances in forecasting cerebral edema, our results detected much better prognostic abilities of neutrophils than NLR for MCE (7). Compared with cerebrum, the differential response manners of cerebellar BBB to inflammatory stimulation may be an important reason for this phenomenon (7). An *in vitro* experiment demonstrated the lower expression of major tight junction components of occludin, claudin-1 and claudin-3 in cerebellar endothelial cells compared with the cerebrum (25). A stronger elevation of VCAM-1 and ICAM-1 expression in cerebellar endothelial cells suggested a higher interaction between inflammatory cells and vessels, which might lead the cerebellum to be more sensitive to neutrophils, the fastest-accumulated cells in infarcted regions after stroke (26). The products of neutrophils including reactive oxide species, proteases (matrix proteinase 3, elastase), lipocalin-2, and neutrophil extracellular traps would directly degrade junction proteins among endothelial cells in BBB (27). The cytokines such as interleukin-1β, interleukin-6, and interleukin-8 from neutrophils also played detrimental roles in BBB integrity and amplified the inflammatory response (28). Taken together, our findings revealed that the neutrophil count was an early independent predictor for the malignant edema of cerebellum and functional outcome in ABAO patients treated by EVT.

This study has several limitations. First, the ABAO patients included in current research were from the national registry of China, and thus, the associations between inflammatory composites and outcomes need to be tested in non-Chinese populations. Second, the acute stress, chemoradiotherapy and the applications of medication, such as corticosteroids and antibiotics, might lead to the modulations of count and functions of neutrophils. We did not assess their impacts on neutrophils but attempted to minimize their effects by only using the baseline neutrophil count at admission and excluding patients with prior medication use and relevant disease history. Third, though initial infarction volumes were reported to be related with brain edema, we did not assess it due to the fact that we included patients through preoperative or postoperative imaging examinations, and there were no preoperative CTP or MRI imaging data for all patients. In addition, the technical differences between CTP and MRI might also introduce a technique bias when assessing infarct volumes (6). To lower the relevant impacts, NIHSS, PC-ASPECTS, collateral circulation status, and onset to treatment time, which were important influential factors for infarct volumes, were included in the present analysis (29, 30). Fourth, recent studies have illustrated the gender differences on the inflammation response. Although our results indicated that there was no significant difference in the prognostic role of neutrophils in ABAO patients, further analysis was limited due to the relatively small number of female patients (31). Due to the real-world design of the present study, we cannot specifically control for gender ratio when enrolling patients. Based on your valuable suggestion, we will strictly control the gender proportion in the national RCT BASILAR2 (Clinical Trial Registry identifier: ChiCTR2000040902), which is being carried out by our group, and try to explore the characteristics of inflammatory response pattern in female ABAO patients in the BASILAR2 trail. Fifth, compared with measuring inflammatory cells once, the dynamic alterations of these factors might provide additional prognostic information. We will try to monitor the longitudinal alterations of inflammatory factors and explore their roles in determining MCE and outcomes in the multi-center random clinical trial of BASILAR2.

## Conclusions

MCE acted essential roles in worsening the prognosis for ABAO after EVT, and thus, additional treatments before, during, and after EVT, such as reducing MCE, were needed to improve clinical outcome for ABAO patients. These 5 factors including the onset to recanalization time, baseline NIHSS score, collateral circulation, neutrophil count at admission, and recanalization status were critical contributors to MCE. Compared with these unchangeable factors including stroke severity and the collateral circulation status, the admission neutrophil count could not only act as an easily accessible biomarker from a blood routine examination to be incorporated into the clinical decision-making system but also be a target to guide immunomodulation therapy for ABAO patients.

## Code Availability

The analyzing codes of this study are available from the corresponding author upon reasonable request.

## Data Availability Statement

The raw data supporting the conclusions of this article will be made available by the authors, without undue reservation.

## Ethics Statement

The studies involving human participants were reviewed and approved by Xinqiao Hospital. The patients/participants provided their written informed consent to participate in this study.

## Author Contributions

CL: Drafting/revision of the manuscript for content, including medical writing for content, major role in the acquisition of data, analysis or interpretation of data. FL: Study concept or design, analysis, or interpretation of data. SL: Study concept or design. QC: Major role in the acquisition of data. HS: Study concept or design, analysis, or interpretation of data. QY: Drafting/revision of the manuscript for content, including medical writing for content, study concept or design. KZ: Drafting/revision of the manuscript for content, including medical writing for content, Study concept or design. WZ: Drafting/revision of the manuscript for content, including medical writing for content, major role in the acquisition of data, study concept or design. All authors contributed to the article and approved the submitted version.

## Funding

This work was supported by National Natural Science Foundation of China (No. 82001264, 82071323, 82001265, 81901236), Chongqing Natural Science Foundation (No. cstc2020jcyj-msxmX0926), Clinical Medical Research Talent Training Program of Army Medical University (No. 2019XLC2008 and 2019XLC3016).

## Conflict of Interest

The authors declare that the research was conducted in the absence of any commercial or financial relationships that could be construed as a potential conflict of interest.

## Publisher’s Note

All claims expressed in this article are solely those of the authors and do not necessarily represent those of their affiliated organizations, or those of the publisher, the editors and the reviewers. Any product that may be evaluated in this article, or claim that may be made by its manufacturer, is not guaranteed or endorsed by the publisher.

## References

[1] Mattle HP Arnold M Lindsberg PJ Schonewille WJ Schroth G. Basilar Artery Occlusion. Lancet Neurol (2011) 10(11):1002–14. doi: 10.1016/S1474-4422(11)70229-0 22014435

[2] Liu X Dai Q Ye R Zi W Liu Y Wang H. Endovascular Treatment Versus Standard Medical Treatment for Vertebrobasilar Artery Occlusion (BEST): An Open-Label, Randomised Controlled Trial. Lancet Neurol (2020) 19(2):115–22. doi: 10.1016/S1474-4422(19)30395-3 31831388

[3] Langezaal LCM van der Hoeven E Mont’Alverne FJA de Carvalho JJF Lima FO Dippel DWJ. Endovascular Therapy for Stroke Due to Basilar-Artery Occlusion. N Engl J Med (2021) 384(20):1910–20. doi: 10.1056/NEJMoa2030297 34010530

[4] Yoon W Baek BH Lee YY Kim SK Kim JT Park MS. Association of Pretreatment Pontine Infarction With Extremely Poor Outcome After Endovascular Thrombectomy in Acute Basilar Artery Occlusion. J Neurointerv Surg (2021) 13(2):136–40. doi: 10.1136/neurintsurg-2020-015930 32447299

[5] Fabritius MP Thierfelder KM Meinel FG Othman AE Dorn F Sabel BO. Early Imaging Prediction of Malignant Cerebellar Edema Development in Acute Ischemic Stroke. Stroke (2017) 48(9):2597–600. doi: 10.1161/STROKEAHA.117.018237 28687640

[6] Mourand I Mahmoudi M Dargazanli C Pavillard F Arquizan C Labreuche J. DWI Cerebellar Infarct Volume as Predictor of Outcomes After Endovascular Treatment of Acute Basilar Artery Occlusion. J Neurointerv Surg (2020) 13(11):995–1001. doi: 10.1136/neurintsurg-2020-016804 33243771

[7] Ferro D Matias M Neto J Dias R Moreira G Petersen N. Neutrophil-to-Lymphocyte Ratio Predicts Cerebral Edema and Clinical Worsening Early After Reperfusion Therapy in Stroke. Stroke (2021) 52(3):859–67. doi: 10.1161/STROKEAHA.120.032130 33517702

[8] Enzmann G Kargaran S Engelhardt B. Ischemia-Reperfusion Injury in Stroke: Impact of the Brain Barriers and Brain Immune Privilege on Neutrophil Function. Ther Adv Neurol Disord (2018) 11:1756286418794184. doi: 10.1177/1756286418794184 30181779 PMC6111395

[9] Strbian D Sairanen T Silvennoinen H Salonen O Kaste M Lindsberg PJ. Thrombolysis of Basilar Artery Occlusion: Impact of Baseline Ischemia and Time. Ann Neurol (2013) 73(6):688–94. doi: 10.1002/ana.23904 23536323

[10] Reinhard M Schork J Allignol A Weiller C Kaube H. Cerebellar and Cerebral Autoregulation in Migraine. Stroke (2012) 43(4):987–93. doi: 10.1161/STROKEAHA.111.644674 22343638

[11] Writing Group for the BG Zi W Qiu Z Wu D Li F Liu H. Assessment of Endovascular Treatment for Acute Basilar Artery Occlusion *Via* a Nationwide Prospective Registry. JAMA Neurol (2020) 77(5):561–73. doi: 10.1001/jamaneurol.2020.0156 PMC7042866 32080711

[12] van der Hoeven EJ McVerry F Vos JA Algra A Puetz V Kappelle LJ. Collateral Flow Predicts Outcome After Basilar Artery Occlusion: The Posterior Circulation Collateral Score. Int J Stroke (2016) 11(7):768–75. doi: 10.1177/1747493016641951 27016515

[13] Jauss M Muffelmann B Krieger D Zeumer H Busse O. A Computed Tomography Score for Assessment of Mass Effect in Space-Occupying Cerebellar Infarction. J Neuroimaging (2001) 11(3):268–71. doi: 10.1111/j.1552-6569.2001.tb00045.x 11462293

[14] von Kummer R Broderick JP Campbell BC Demchuk A Goyal M Hill MD. The Heidelberg Bleeding Classification: Classification of Bleeding Events After Ischemic Stroke and Reperfusion Therapy. Stroke (2015) 46(10):2981–6. doi: 10.1161/STROKEAHA.115.010049 26330447

[15] Zhang SR Phan TG Sobey CG. Targeting the Immune System for Ischemic Stroke. Trends Pharmacol Sci (2021) 42(2):96–105. doi: 10.1016/j.tips.2020.11.010 33341247

[16] Broocks G Elsayed S Kniep H Kemmling A Flottmann F Bechstein M. Early Prediction of Malignant Cerebellar Edema in Posterior Circulation Stroke Using Quantitative Lesion Water Uptake. Neurosurgery (2021) 88(3):531–7. doi: 10.1093/neuros/nyaa438 33040147

[17] Mourand I Machi P Nogue E Arquizan C Costalat V Picot MC. Diffusion-Weighted Imaging Score of the Brain Stem: A Predictor of Outcome in Acute Basilar Artery Occlusion Treated With the Solitaire FR Device. AJNR Am J Neuroradiol (2014) 35(6):1117–23. doi: 10.3174/ajnr.A3870 PMC7965148 24524920

[18] Wang P Jia X Zhang M Cao Y Zhao Z Shan Y. Correlation of Longitudinal Gray Matter Volume Changes and Motor Recovery in Patients After Pontine Infarction. Front Neurol (2018) 9:312. doi: 10.3389/fneur.2018.00312 29910762 PMC5992285

[19] Maestrini I Strbian D Gautier S Haapaniemi E Moulin S Sairanen T. Higher Neutrophil Counts Before Thrombolysis for Cerebral Ischemia Predict Worse Outcomes. Neurology (2015) 85(16):1408–16. doi: 10.1212/WNL.0000000000002029 PMC4626239 26362283

[20] Boisseau W Desilles JP Fahed R Kyheng M Zuber K Sabben C. Neutrophil Count Predicts Poor Outcome Despite Recanalization After Endovascular Therapy. Neurology (2019) 93(5):e467–e75. doi: 10.1212/WNL.0000000000007859 31239356

[21] Chen C Huang T Zhai X Ma Y Xie L Lu B. Targeting Neutrophils as a Novel Therapeutic Strategy After Stroke. J Cereb Blood Flow Metab (2021) 41(9):2150–61. doi: 10.1177/0271678X211000137 PMC8393299 33691513

[22] Cai W Liu S Hu M Huang F Zhu Q Qiu W. Functional Dynamics of Neutrophils After Ischemic Stroke. Transl Stroke Res (2020) 11(1):108–21. doi: 10.1007/s12975-019-00694-y PMC6993940 30847778

[23] Wanrooy BJ Wen SW Wong CH. Dynamic Roles of Neutrophils in Post-Stroke Neuroinflammation. Immunol Cell Biol (2021) 99(9):924–35. doi: 10.1111/imcb.12463 33894069

[24] Qiu YM Zhang CL Chen AQ Wang HL Zhou YF Li YN. Immune Cells in the BBB Disruption After Acute Ischemic Stroke: Targets for Immune Therapy? Front Immunol (2021) 12:678744. doi: 10.3389/fimmu.2021.678744 34248961 PMC8260997

[25] Silwedel C Forster C. Differential Susceptibility of Cerebral and Cerebellar Murine Brain Microvascular Endothelial Cells to Loss of Barrier Properties in Response to Inflammatory Stimuli. J Neuroimmunol (2006) 179(1-2):37–45. doi: 10.1016/j.jneuroim.2006.06.019 16884785

[26] Engelhardt B Ransohoff RM. The Ins and Outs of T-lymphocyte Trafficking to the CNS: Anatomical Sites and Molecular Mechanisms. Trends Immunol (2005) 26(9):485–95. doi: 10.1016/j.it.2005.07.004 16039904

[27] Ludewig P Sedlacik J Gelderblom M Bernreuther C Korkusuz Y Wagener C. Carcinoembryonic Antigen-Related Cell Adhesion Molecule 1 Inhibits MMP-9-mediated Blood-Brain-Barrier Breakdown in a Mouse Model for Ischemic Stroke. Circ Res (2013) 113(8):1013–22. doi: 10.1161/CIRCRESAHA.113.301207 23780386

[28] Soehnlein O Steffens S Hidalgo A Weber C. Neutrophils as Protagonists and Targets in Chronic Inflammation. Nat Rev Immunol (2017) 17(4):248–61. doi: 10.1038/nri.2017.10 28287106

[29] Al-Dasuqi K Payabvash S Torres-Flores GA Strander SM Nguyen CK Peshwe KU. Effects of Collateral Status on Infarct Distribution Following Endovascular Therapy in Large Vessel Occlusion Stroke. Stroke (2020) 51(9):e193–202. doi: 10.1161/STROKEAHA.120.029892 PMC7484023 32781941

[30] Cheng B Forkert ND Zavaglia M Hilgetag CC Golsari A Siemonsen S. Influence of Stroke Infarct Location on Functional Outcome Measured by the Modified Rankin Scale. Stroke (2014) 45(6):1695–702. doi: 10.1161/STROKEAHA.114.005152 24781084

[31] Rexrode KM Madsen TE Yu AYX Carcel C Lichtman JH Miller EC. The Impact of Sex and Gender on Stroke. Circ Res (2022) 130(4):512–28. doi: 10.1161/CIRCRESAHA.121.319915 PMC8890686 35175851

