# Supplementary material for: Neutrophil Count Predicts Malignant Cerebellar Edema and Poor Outcome in Acute Basilar Artery Occlusion Receiving Endovascular Treatment: A Nationwide Registry-Based Study
Source: Front Immunol. 2022 May 3;13:835915. doi: 10.3389/fimmu.2022.835915 (PMC9111017; doi:10.3389/fimmu.2022.835915)
Supplement: Supplementary file 1 [file DataSheet_1.docx]

**Supplemental Table 1. Clinical features for enrolled patients stratified by different long-term functional outcome.**

|  | **All patients**  **(N=329)** | **mRS≤3**  **(N=89)** | **mRS>3**  **(N=240)** | **P** |
| --- | --- | --- | --- | --- |
| Age, years, mean±SD | 63.86±10.83 | 63.22±11.22 | 64.10±10.70 | 0.516 |
| Men, (n%) | 257 (78.12) | 64 (71.91) | 193 (80.42) | 0.097 |
| Baseline NIHSS, median(IQR) | 27.00(17.00, 33.00) | 17.00(8.00, 25.00) | 30.00(21.00, 34.00) | <0.001 |
| Initial PC-ASPECTS, median(IQR) | 8.00(6.00, 9.00) | 8.00(8.00, 10.00) | 7.00(6.00, 8.00) | <0.001 |
| Admission SBP, mmHg, mean±SD | 150.97±25.79 | 149.27±24.51 | 151.60±26.27 | 0.468 |
| Admission DBP, mmHg, mean±SD | 86.74±15.67 | 85.26±13.91 | 87.28±16.27 | 0.299 |
| 24h NIHSS after EVT, median(IQR) | 29.00(15.00, 35.00) | 9.00(2.00, 17.00) | 32.00(26.00, 36.00) | <0.001 |
| 7d NIHSS after EVT, median(IQR) | 24.00(9.00, 36.00) | 3.00(1.00, 7.00) | 34.00(21.75, 36.00) | <0.001 |
| Intravenous thrombolysis | 71 (21.58) | 16 (17.98) | 55 (22.92) | 0.333 |
| Pre-onset mRS |  |  |  |  |
| 0 | 284 (86.32) | 79 (88.76) | 205 (85.42) | 0.228 |
| 1 | 31 (9.42) | 9 (10.11) | 22 (9.17) |  |
| 2 | 14 (4.26) | 1 (1.12) | 13 (5.42) |  |
| Decompressive craniectomy, (n%) | 11(3.34) | 1(1.12) | 10(4.17) | 0.300 |
| History of risk factors, n(%) | |  |  |  |
| Hypertension | 231 (70.21) | 65 (73.03) | 166 (69.17) | 0.496 |
| Diabetes mellitus | 75 (22.80) | 15 (16.85) | 60 (25.00) | 0.118 |
| Dylipidemia | 109 (33.13) | 33(37.08) | 76 (31.67) | 0.354 |
| Atrial fibrillation | 59 (17.93) | 18 (20.22) | 41 (17.08) | 0.509 |
| TIA | 3 (0.91) | 0 (0.00) | 3 (1.25) | 0.289 |
| Laboratory results, median(IQR) |  |  |  |  |
| Neutrophil,10^9^/L | 9.87(7.28, 12.40) | 7.83(6.00,9.90) | 10.78(8.12,13.34) | <0.001 |
| Lymphocyte, 10^9^/L | 1.09(0.80, 1.60) | 1.20(0.82,1.65) | 1.04(0.80,1.55) | 0.133 |
| NLR | 8.83(5.35, 13.66) | 6.44(3.89,9.89) | 9.83(5.94,14.39) | 0.001 |
| Platelet count, 10^9^/L | 216.00(177.00,250.00) | 207.00(173.00,241.00) | 218.00(177.00,253.75) | <0.001 |
| PLR | 180.83(131.09, 277.61) | 165.48(123.48,225.23) | 193.65(134.79, 286.23) | 0.007 |
| TOAST classification, n(%) | |  |  | 0.334 |
| LAA | 227(69.00) | 56(62.92) | 171(71.25) |  |
| CE | 73(22.19) | 25(28.09) | 48(20.00) |  |
| SOE | 7(2.13) | 1(1.12) | 6(2.50) |  |
| SUE | 22(6.69) | 7(7.87) | 15(6.25) |  |
| Imaging parameters |  |  |  |  |
| Occlusion site, n(%) |  |  |  | 0.004 |
| BA distal | 96 (29.18) | 39 (43.82) | 57 (23.75) |  |
| BA middle | 108 (32.83) | 25 (28.09) | 83 (34.58) |  |
| BA proximal | 56 (17.02) | 13 (14.61) | 43 (17.92) |  |
| V4 | 69 (20.97) | 12 (13.48) | 57 (23.75) |  |
| PC-CS score, median(IQR) | 4.00(3.00, 6.00) | 5.00(4.00, 6.00) | 4.00(3.00, 5.00) | <0.001 |
| Reperfusion status, n(%) | |  |  |  |
| mTICI |  |  |  | <0.001 |
| 0-2a | 60(18.24) | 4(4.49) | 56(23.33) |  |
| 2b-3^a^ | 269(81.76) | 85(95.51) | 184(76.67) |  |
| Treatment delay, median(IQR), min | |  |  |  |
| Onset to puncture | 334.00(219.75, 497.00) | 270.00(200.00, 413.00) | 360.00(239.50, 506.00) | 0.028 |
| Puncture to recanalization | 107.00(72.00, 152.25) | 91.00(61.00, 126.00) | 115.00(78.00, 164.00) | <0.001 |
| Onset to recanalization | 456.00(327.75, 633.00) | 385.00(299.00, 508.00) | 480.00(357.00, 652.50) | 0.005 |
| Severe adverse events, n(%) |  |  |  |  |
| MCE status |  |  |  | <0.001 |
| Non-MCE | 210(63.83) | 77(86.52) | 133(55.42) |  |
| MCE | 119(36.17) | 12(13.48) | 107(44.58) |  |
| Any hemorrhage | 41(12.46) | 3(3.37) | 38(15.83) | 0.001 |
| sICH | 31(9.42) | 2(2.25) | 29(12.08) | 0.005 |

^a^mTICI score of 2b or 3 indicates complete recanalization.

PC-CS, posterior circulation collateral system score; BA, basilar artery; mTICI, modified thrombolysis in cerebral infarction; PCA, posterior cerebral artery; V4, V4 segment of vertebral artery; CE, cardioembolism; NIHSS, National Institutes of Health Stroke Scale; PC-ASPECTS, posterior circulation Alberta Stroke Program Early CT Score; SBP, systolic blood pressure; DBP, diastolic blood pressure; SOE, stroke of other determined cause; SUE, stroke of undetermined cause; TIA, transient ischemic attack; TOAST, Trial of ORG 10172 in Acute Stroke Treatment.

**Supplemental Table 2: Clinical features for enrolled patients stratified by different neutrophil status.**

|  | **All patients**  **(N=329)** | **High neutrophil counts(N=164)** | **Low neutrophil couts(N=165)** | **P** |
| --- | --- | --- | --- | --- |
| Age, years, mean±SD | 63.86 ±10.83 | 62.88(11.56) | 64.84(10.00) | 0.100 |
| Men, (n%) | 257(78.12) | 130(79.27) | 127(76.97) | 0.614 |
| Baseline NIHSS, median(IQR) | 27.00(17.00,33.00) | 28.00(20.00,34.00) | 25.00(14.00,32.00) | 0.011 |
| Initial PC-ASPECTS, median(IQR) | 8.00(6.00,9.00) | 8.00(6.00,9.00) | 8.00(7.00-9.00) | 0.293 |
| Admission SBP, mmHg, mean±SD | 150.97±25.79 | 151.43±23.93 | 150.51±27.58 | 0.747 |
| Admission DBP, mmHg, mean±SD | 86.74±15.67 | 86.71±16.33 | 86.76±15.04 | 0.980 |
| 24h NIHSS after EVT, median(IQR) | 29.00(15.00,35.00) | 32.00(21.75,36.00) | 22.00(10.00,33.00) | <0.001 |
| 7d NIHSS after EV, median(IQR) | 24.00(9.00,36.00) | 32.00(17.00,36.00) | 17.00 (5.00,35.00) | <0.001 |
| Intravenous thrombolysis, (n%) | 71(21.58) | 33(20.12) | 38(23.03) | 0.521 |
| Pre-onset mRS, (n%) |  |  |  |  |
| 0 | 284(86.32) | 141(85.98) | 143 (86.67) | 0.979 |
| 1 | 31(9.42) | 16(9.76) | 15 (9.09) |  |
| 2 | 14(4.26) | 7(4.27) | 7(4.24) |  |
| Decompressive craniectomy, (n%) | 11(3.34) | 5(3.05) | 6(3.63) | 0.767 |
| History of risk factors, n(%) | |  |  |  |
| Hypertension | 231(70.21) | 115(70.12) | 116(70.30) | 0.971 |
| Diabetes mellitus | 75(22.80) | 35(21.34) | 40(24.24) | 0.531 |
| Dylipidemia | 109 (33.13) | 59(35.98) | 50(30.30) | 0.274 |
| Atrial fibrillation | 59(17.93) | 28(17.07) | 31(18.79) | 0.685 |
| TIA | 3(0.91) | 2(1.22) | 1(0.61) | 0.558 |
| Laboratory results, median(IQR) |  |  |  |  |
| Neutrophil, 10^9^/L | 9.87(7.28,12.40) | 12.43(11.06,14.57) | 7.28(5.68,8.70) | <0.001 |
| Lymphocyte, 10^9^/L | 1.09(0.80,1.60) | 1.02(0.80,1.41) | 1.19(0.82,1.73) | 0.024 |
| NLR | 8.83(5.35,13.66) | 12.73(8.47,16.45) | 5.85(3.56,9.55) | <0.001 |
| Platelet count, 10^9^/L | 216.00(177.00,250.00) | 223.00(186.00,270.75) | 204.00(165.00,239.00) | <0.001 |
| PLR | 180.83(131.09,277.61) | 198.78(147.70,303.57) | 156.43(115.46,241.03) | <0.001 |
| TOAST classification, n(%) | |  |  | 0.776 |
| LAA | 227(69.00) | 116(70.73) | 111(67.27) |  |
| CE | 73(22.19) | 33(20.12) | 40(24.24) |  |
| SOE | 7(2.13) | 3(1.83) | 4(2.42) |  |
| SUE | 22(6.69) | 12(7.32) | 10(6.06) |  |
| Imaging parameters |  |  |  |  |
| Occlusion site, n(%) |  |  |  | 0.058 |
| BA distal | 96(29.18) | 40(24.39) | 56(33.94) |  |
| BA middle | 108(32.83) | 51(31.10) | 57(34.55) |  |
| BA proximal | 56(17.02) | 30(18.29) | 26(15.76) |  |
| V4 | 69(20.97) | 43(26.22) | 26(15.76) |  |
| PC-CS score, median(IQR) | 4.00(3.00,6.00) | 4.00(3.00,6.00) | 4.00(3.00,6.00) | 0.473 |
| Reperfusion status, n(%) | |  |  |  |
| mTICI |  |  |  | 0.146 |
| 0-2a | 60(18.24) | 35(21.34) | 25(15.15) |  |
| 2b-3^a^ | 269(80.78) | 129(78.66) | 140(84.84) |  |
| Treatment delay, min, median(IQR) | |  |  |  |
| Onset to puncture | 334.00(219.75, 497.00) | 336.00(231.50,507.50) | 330.00(211.00,494.65) | 0.410 |
| Puncture to recanalization | 107.00(72.00,152.25) | 119.00(79.50,159.00) | 101.00(65. 25,141.00) | 0.004 |
| Onset to recanalization | 456.00(327.75,633.00) | 469.00(351.19,652.50) | 443.00(320.00,602.00) | 0.119 |
| Severe adverse events, n(%) |  |  |  |  |
| MCE | 119(36.17) | 81(49.39) | 38(23.03) | <0.001 |
| Any hemorrhage | 41(12.46) | 24(14.63) | 17(10.30) | 0.234 |
| sICH | 31(9.57) | 18(11.25) | 13(7.93) | 0.309 |

^a^mTICI score of 2b or 3 indicates complete recanalization.

PC-CS, posterior circulation collateral system score; BA, basilar artery; mTICI, modified thrombolysis in cerebral infarction; PCA, posterior cerebral artery; V4, V4 segment of vertebral artery; CE, cardioembolism; NIHSS, National Institutes of Health Stroke Scale; PC-ASPECTS, posterior circulation Alberta Stroke Program Early CT Score; SBP, systolic blood pressure; DBP, diastolic blood pressure; SOE, stroke of other determined cause; SUE, stroke of undetermined cause; TIA, transient ischemic attack; TOAST, Trial of ORG 10172 in Acute Stroke Treatment.

**Supplemental Table 3. Sensitive analysis for the roles of neutrophil on cohorts after exclusion of symptomatic hemorrhagic, transformation, and unsuccessful revascularization.**

|  | **Exclude sICH cohorts** | | **Exclude hemorrhage cohorts** | | | **Satisfied reperfusion cohorts** | | |
| --- | --- | --- | --- | --- | --- | --- | --- | --- |
|  | **Adjusted OR(95%CI)** | **P value** | | **Adjusted OR(95%CI)** | **P** | | **Adjusted OR(95%CI)** | **P** |
| MCE | 2.65(1.51,4.71) ^c^ | <0.001 | | 2.63(1.45, 4.85) ^c^ | 0.002 | | 2.60(1.46,4.68) ^c^ | 0.001 |
|  |  |  | |  |  | |  |  |
| 90 day-mRS 0-3, n(%) | 0.32(0.15,0.60) ^c^ | <0.001 | | 0.36 (0.18-0.72) ^c^ | 0.004 | | 0.30(0.14,0.59) ^c^ | <0.001 |
|  |  |  | |  |  | |  |  |
| mRS, median(IQR) | 2.00(1.24-3.23) ^d^ | 0.005 | | 2.03(1.23,3.36) ^d^ | 0.006 | | 2.12(1.31,3.46) ^d^ | 0.002 |
|  |  |  | |  |  | |  |  |
| Mortality in hospital, n(%) | 1.04(0.53,2.02) ^c^ | 0.916 | | 1.09(0.55,2.20) ^c^ | 0.799 | | 1.70(0.84,3.50) ^c^ | 0.144 |
|  |  |  | |  |  | |  |  |
| Mortality at 90d, n(%) | 1.92(1.09,3.39) ^c^ | 0.024 | | 1.93(1.06,3.55) ^c^ | 0.032 | | 2.09(1.19,3.72) ^c^ | 0.011 |
|  |  |  | |  |  | |  |  |
| ΔNIHSS at 24 h ^a^ ,  median(IQR) | 3.31(1.27 to 5.35) ^e^ | 0.002 | | 2.66(0.56 to 4.75)^e^ | 0.013 | | 3.53(1.37 to 5.68)^e^ | 0.001 |
|  |  |  | |  |  | |  |  |
| ΔNIHSS at 5-7d ^b^,  median(IQR) | 4.02(1.48 to 6.57) ^e^ | 0.002 | | 3.54(0.91 to 6.17)^e^ | 0.009 | | 4.69(1.96 to 7.43)^e^ | <0.001 |

^a^ Change from NIHSS at admission from NIHSS at 24 hours after EVT.

^b^ Change from NIHSS at admission from NIHSS at 5-7 days after EVT

^c^Adjusted odds ratio; adjusted estimates of outcome were calculated using multiple regression, taking the following variables into account: baseline NIHSS score, baseline PC-ASPECTS, neutrophil count, mTICI, PC-CS score, occlusion sites, and onset to recanalization time.

^d^Adjusted common odds ratio; adjusted estimates of outcome were calculated using multiple regression, taking the following variables into account: baseline NIHSS score, baseline PC-ASPECTS, neutrophil count, mTICI, PC-CS score, occlusion sites, and onset to recanalization time.

^e^ß-values were estimated from a multivariable linear regression model; adjusted estimates of outcome were calculated using multiple regression, taking the following variables into account: baseline NIHSS score, baseline PC-ASPECTS, neutrophil count, mTICI, PC-CS score, occlusion sites, and onset to recanalization time.

NIHSS, National Institutes of Health Stroke Scale; mRS, modified Rankin Scale score at 90 days; MCE, malignant cerebellar edema; PC-CS, posterior circulation collateral system score; mTICI, modified thrombolysis in cerebral infarction; PC-ASPECTS, posterior circulation Alberta Stroke Program Early CT Score;

**Supplemental Table 4. The predictive roles of neutrophil status for outcome among all ABAO patients after EVT in BASILAR registry.**

|  | **Low Neu**  **group** | **High Neu**  **group** | **P value** | **OR(95%CI)** | **P** | **Adjusted OR(95%CI)** | **P** |
| --- | --- | --- | --- | --- | --- | --- | --- |
| 90 day-mRS 0-3, n(%) | 133(40.06) | 56(22.05) | <0.001^c^ | 0.42(0.29,0.60) | <0.001 | 0.49(0.31-0.78)^g^ | 0.002 |
|  |  |  |  |  |  |  |  |
| mRS, median(IQR) | 5.00(1.00, 6.00) | 6.00(4.00, 6.00) | <0.001^d^ | 1.95(1.36,2.81)^e^ | <0.001 | 1.51(1.09,2.11)^h^ | 0.015 |
|  |  |  |  |  |  |  |  |
| Mortality in hospital, n(%) | 61(18.37) | 63(24.80) | 0.059^c^ | 1.45(0.98,2.16) | 0.066 | 1.13(0.73,1.77)^g^ | 0.584 |
|  |  |  |  |  |  |  |  |
| Mortality at 90d, n(%) | 130(39.16) | 131(51.57) | 0.003^c^ | 1.63(1.18,2.27) | 0.004 | 1.33(0.90,1.99)^g^ | 0.156 |
|  |  |  |  |  |  |  |  |
| ΔNIHSS at 24 h ^a^ ,  median(IQR) | 4.00(-10.00, 3.00) | 0.00(10.00, 6.00) | 0.004^d^ | 2.33(0.69 to 3.97)^f^ | 0.005 | 2.30(0.80 to 3.80)^i^ | 0.003 |
|  |  |  |  |  |  |  |  |
| ΔNIHSS at 5-7d ^b^,  median(IQR) | 0.00(-5.00, 2.00) | 0.00(-2.00, 4.00) | 0.001^d^ | 2.98(0.89 to 5.07)^f^ | 0.005 | 2.76(0.93 to 4.60)^i^ | 0.003 |
|  |  |  |  |  |  |  |  |

^a^ Change from NIHSS at admission from NIHSS at 24 hours after EVT.

^b^ Change from NIHSS at admission from NIHSS at 5-7 days after EVT

^c^Chi-square test.

^d^Wilcoxon test.

^e^Common odds ratio.

^f^ß-values were estimated from a univariate linear regression model.

^g^Adjusted odds ratio; adjusted estimates of outcome were calculated using multiple regression, taking the following variables into account: baseline NIHSS score, baseline PC-ASPECTS, neutrophil count, mTICI, PC-CS score, occlusion sites, and onset to recanalization time.

^h^Adjusted common odds ratio; adjusted estimates of outcome were calculated using multiple regression, taking the following variables into account: baseline NIHSS score, baseline PC-ASPECTS, neutrophil count, mTICI, PC-CS score, occlusion sites, and onset to recanalization time.

^i^ß-values were estimated from a multivariable linear regression model; adjusted estimates of outcome were calculated using multiple regression, taking the following variables into account: baseline NIHSS score, baseline PC-ASPECTS, neutrophil count, mTICI, PC-CS score, occlusion sites, and onset to recanalization time.

NIHSS, National Institutes of Health Stroke Scale; mRS, modified Rankin Scale score at 90 days; MCE, malignant cerebellar edema; PC-CS, posterior circulation collateral system score; mTICI, modified thrombolysis in cerebral infarction; PC-ASPECTS, posterior circulation Alberta Stroke Program Early CT Score;

**Supplemental Table 5. Baseline clinical features for normal range group (2-8 x10(9)/L), elevated group (8-9.87 x10(9)/L), and extremely high group(>9.87 x10(9)/L).**

|  | **Normal group**  **(N=104)** | **Elevated group**  **(N=61)** | **Extremely high group**  **(N=164)** | **P** |
| --- | --- | --- | --- | --- |
| Age, years, mean±SD | 65.52±9.41 | 63.69±10.92 | 62.88±11.56 | 0.149 |
| Men, (n%) | 80 (76.92) | 47 (77.05) | 130 (79.27) | 0.880 |
| Baseline NIHSS, median(IQR) | 22.00(13.00,32.00) | 27.00(20.00,32.25) | 28.00(20.00,34.00) | 0.013 |
| Initial PC-ASPECTS, median(IQR) | 8.00(7.00,9.00) | 7.00(6.00,8.00) | 8.00(6.00,9.00) | 0.002 |
| Admission SBP, mmHg, mean±SD | 150.52±25.08 | 150.49±31.60 | 151.43±23.93 | 0.950 |
| Admission DBP, mmHg, mean±SD | 87.07±13.68 | 86.23±17.23 | 86.71±16.33 | 0.947 |
| 24h NIHSS after EVT, median(IQR) | 19.50(7.75,33.00) | 26.50(15.00,35.00) | 32.00(21.75,36.00) | <0.001 |
| 7d NIHSS after EVT, median(IQR) | 13.00(2.75,33.00) | 20.00(11.00,35.00) | 32.00(17.00,36.00) | <0.001 |
| Intravenous thrombolysis | 28 (26.92) | 10 (16.39) | 33 (20.12) | 0.231 |
| Pre-onset mRS |  |  |  | 0.794 |
| 0 | 89 (85.58) | 54 (88.52) | 141 (85.98) |  |
| 1 | 9 (8.65) | 6 (9.84) | 16 (9.76) |  |
| 2 | 6 (5.77) | 1 (1.64) | 7 (4.27) |  |
| Decompressive craniectomy, (n%) | 4(3.85) | 2(3.33) | 5(3.03) | 0.939 |
| History of risk factors, n(%) | |  |  |  |
| Hypertension | 76 (73.08) | 40 (65.57) | 115 (70.12) | 0.596 |
| Diabetes mellitus | 25 (24.04) | 15 (24.59) | 35 (21.34) | 0.819 |
| Dylipidemia | 31(29.81) | 19(31.15) | 59(35.98) | 0.735 |
| Atrial fibrillation | 21 (20.19) | 10 (16.39) | 28(17.07) | 0.763 |
| TIA | 1 (0.96) | 0 (0.00) | 2(1.22) | 0.692 |
| Laboratory results, median(IQR) |  |  |  |  |
| Neutrophil,10^9^/L | 6.29(4.76,7.00) | 9.00(8.54,9.50) | 12.43(11.06,14.57) | <0.001 |
| Lymphocyte, 10^9^/L | 1.23(0.93,1.80) | 1.00(0.78,1.60) | 1.02(0.80,1.41) | 0.023 |
| NLR | 4.43(3.14,7.33) | 8.38(5.49,11.86) | 12.73(8.47,16.45) | <0.001 |
| Platelet count, 10^9^/L | 201.50(153.75,237.50) | 210.00(176.00,243.00) | 223.00(186.00,270.75) | <0.001 |
| TOAST classification, n(%) | |  |  | 0.389 |
| LAA | 69 (66.35) | 42 (68.85) | 116 (70.73) |  |
| CE | 29 (27.88) | 11 (18.03) | 33 (20.12) |  |
| SOE | 1 (0.96) | 3 (4.92) | 3 (1.83) |  |
| SUE | 5 (4.81) | 5 (8.20) | 12 (7.32) |  |
| Imaging parameters |  |  |  |  |
| Occlusion site, n(%) |  |  |  | 0.036 |
| BA distal | 41(39.42) | 15 (24.59) | 40(24.39) |  |
| BA middle | 31(29.81) | 26 (42.62) | 51(31.10) |  |
| BA proximal | 14(13.46) | 12(19.67) | 30(18.29) |  |
| V4 | 18(17.31) | 8(13.11) | 43(26.22) |  |
| PC-CS score, median(IQR) | 4.50(3.00,6.00) | 4.00(3.00,6.00) | 4.00(3.00,6.00) | 0.554 |
| Reperfusion status, n(%) | |  |  |  |
| mTICI |  |  |  | 0.102 |
| 0-2a | 12(11.54) | 13(19.67) | 35(7.32) |  |
| 2b-3^a^ | 92(88.46) | 48(78.69) | 129(78.66) |  |
| Treatment delay, median(IQR), min | |  |  |  |
| Onset to puncture | 292.00(201.75,481.00) | 384.00(266.00,595.00) | 336.00(231.50,507.50) | 0.070 |
| Puncture to recanalization | 94.50(63.37,138.75) | 108.00(75.00,141.00) | 119.00(79.50,159.00) | 0.006 |
| Onset to recanalization | 407.00(299.50,575.00) | 497.00(370.00,705.00) | 469.00(351.19,652.50) | 0.026 |
| Severe adverse events, n(%) |  |  |  |  |
| MCE | 24 (23.08) | 14 (22.95) | 81 (49.39) | <0.001 |
| Any hemorrhage | 10 (9.62) | 7 (11.48) | 24 (14.63) | 0.464 |
| sICH | 7 (6.80) | 6 (9.84) | 18 (11.25) | 0.486 |

^a^mTICI score of 2b or 3 indicates complete recanalization.

PC-CS, posterior circulation collateral system score; BA, basilar artery; mTICI, modified thrombolysis in cerebral infarction; PCA, posterior cerebral artery; V4, V4 segment of vertebral artery; CE, cardioembolism; NIHSS, National Institutes of Health Stroke Scale; PC-ASPECTS, posterior circulation Alberta Stroke Program Early CT Score; SBP, systolic blood pressure; DBP, diastolic blood pressure; SOE, stroke of other determined cause; SUE, stroke of undetermined cause; TIA, transient ischemic attack; TOAST, Trial of ORG 10172 in Acute Stroke Treatment.

| **Characteristics** | **Groups** | **No./No.(%)** | **P value** | **OR(95%CI)** | **P** | **Adjusted OR(95%CI)** | **P** |
| --- | --- | --- | --- | --- | --- | --- | --- |
|  | Normal | 46 (44.23) | <0.001^c^ | Reference | Reference | Reference | Reference |
| 90-day mRS 0-3 | Elevated | 19 (31.15) |  | 0.58(0.30, 1.13) | 0.115 | 1.39(0.59, 3.30)^g^ | 0.448 |
|  | Extremely high | 24 (14.63) |  | 0.21(0.12, 0.38) | <0.001 | 0.37(0.18, 0.75) | 0.006 |
|  |  |  |  |  |  |  |  |
|  | Normal | 24(23.08) | <0.001^c^ | Reference | Reference | Reference | Reference |
| MCE | Elevated | 14(22.95) |  | 1.01(0.47, 2.13) | 0.970 | 0.61(0.26, 1.38)^g^ | 0.239 |
|  | Extremely high | 81(49.39) |  | 3.21(1.88, 5.65) | <0.001 | 2.36(1.29, 4.40) | 0.006 |
|  |  |  |  |  |  |  |  |
|  | Normal | 4.00(1.00, 6.00) | <0.001^d^ | Reference | Reference | Reference | Reference |
| mRS, median (IQR) | Elevated | 5.00 (3.00, 6.00) |  | 1.71(0.96, 3.07)^e^ | 0.070 | 0.78(0.42, 1.47)^h^ | 0.44*7* |
|  | Extremely high | 6.00 (5.00, 6.00) |  | 3.29(2.07, 5.26)^e^ | <0.001 | 1.75(1.05, 2.93)^h^ | 0.032 |
|  |  |  |  |  |  |  |  |
|  | Normal | 17 (16.35) | 0.120^c^ | Reference | Reference | Reference | Reference |
| Mortality in hospital | Elevated | 10 (16.39) |  | 1.02(0.42, 2.37) | 0.958 | 0.63(0.23, 1.60)^g^ | 0.343 |
|  | Extremely high | 42 (25.61) |  | 1.75(0.95,3.34) | 0.081 | 1.13(0.57, 2.30)^g^ | 0.720 |
|  |  |  |  |  |  |  |  |
|  | Normal | 36 (34.62) | <0.001^c^ | Reference | Reference | Reference | Reference |
| Mortality at 90d | Elevated | 28 (45.90) |  | 1.65(0.86, 3.17) | 0.129 | 0.88(0.40, 1.94)^g^ | 0.760 |
|  | Extremely high | 97 (59.15) |  | 2.69(1.63,4.52) | <0.001 | 1.60(0.87, 2.98)^g^ | 0.130 |
|  |  |  |  |  |  |  |  |
|  | Normal | -2.00(-12.25, 3.00) | 0.002^d^ | Reference | Reference | Reference | Reference |
| ΔNIHSS at 24h ^b^,  median(IQR) | Elevated | 0.00(-8.00, 2.00) |  | 0.72(-2.36 to 5.10)^f^ | 0.470 | -0.22(-3.80 to 3.05)^i^ | 0.830 |
|  | Extremely high | 0.00 (-6.50, 6.00) |  | 3.28(1.93 to 7.70)^f^ | 0.001 | 2.68(0.97 to 6.38)^i^ | 0.008 |
|  |  |  |  |  |  |  |  |
|  | Normal | 0.00(-4.25, 2.00) | 0.002^d^ | Reference | Reference | Reference | Reference |
| ΔNIHSS at 5-7^a^ ,  median(IQR) | Elevated | 0.00(-5.00, 2.00) |  | 0.50(-2.22 to 3.73)^f^ | 0.618 | -0.32(-3.19 to 2.30)^i^ | 0.747 |
|  | Extremely high | 0.00(0.00, 5.25) |  | 3.16(1.39 to 5.99)^f^ | 0.007 | 2.83(0.95 to 5.29)^i^ | 0.005 |

**Supplemental Table 6. The impacts of neutrophil counts on clinical outcome counts among normal range group (2-8 x10(9)/L), elevated group (8-9.87 x10(9)/L), and extremely high group (>9.87 x10(9)/L).**

^a^ Change from NIHSS at admission from NIHSS at 24 hours after EVT.

^b^ Change from NIHSS at admission from NIHSS at 5-7 days after EVT

^c^Chi-square test.

^d^Wilcoxon test.

^e^Common odds ratio.

^f^ß-values were estimated from a univariate linear regression model.

^g^Adjusted odds ratio; adjusted estimates of outcome were calculated using multiple regression, taking the following variables into account: baseline NIHSS score, baseline PC-ASPECTS, neutrophil count, mTICI, PC-CS score, occlusion sites, and onset to recanalization time.

^h^Adjusted common odds ratio; adjusted estimates of outcome were calculated using multiple regression, taking the following variables into account: baseline NIHSS score, baseline PC-ASPECTS, neutrophil count, mTICI, PC-CS score, occlusion sites, and onset to recanalization time.

^i^ß-values were estimated from a multivariable linear regression model; adjusted estimates of outcome were calculated using multiple regression, taking the following variables into account: baseline NIHSS score, baseline PC-ASPECTS, neutrophil count, mTICI, PC-CS score, occlusion sites, and onset to recanalization time.

NIHSS, National Institutes of Health Stroke Scale; mRS, modified Rankin Scale score at 90 days; MCE, malignant cerebellar edema; PC-CS, posterior circulation collateral system score; mTICI, modified thrombolysis in cerebral infarction; PC-ASPECTS, posterior circulation Alberta Stroke Program Early CT Score;


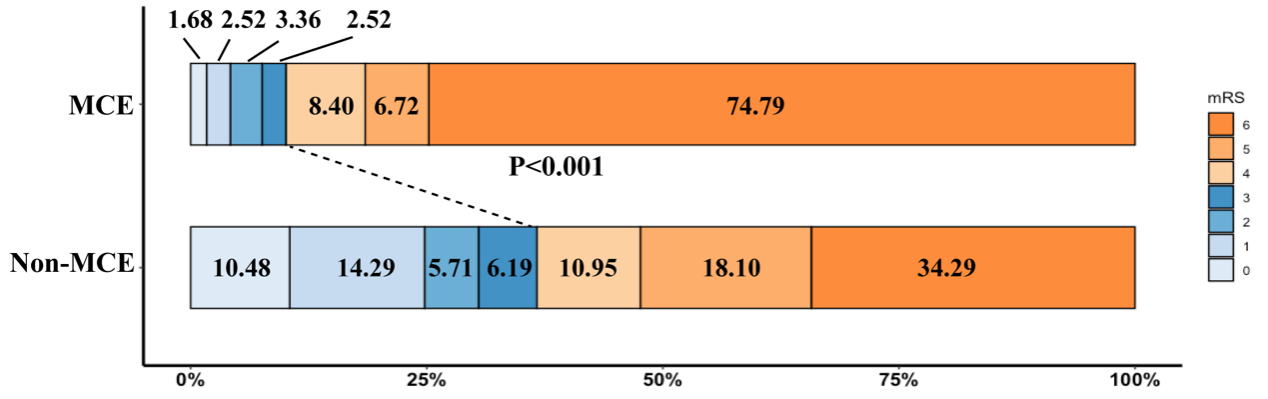


**Supplemental Figure 1.** Distribution of the modified Rankin Scale (mRS) scores at 90 days according to the trichotomized MCE status.


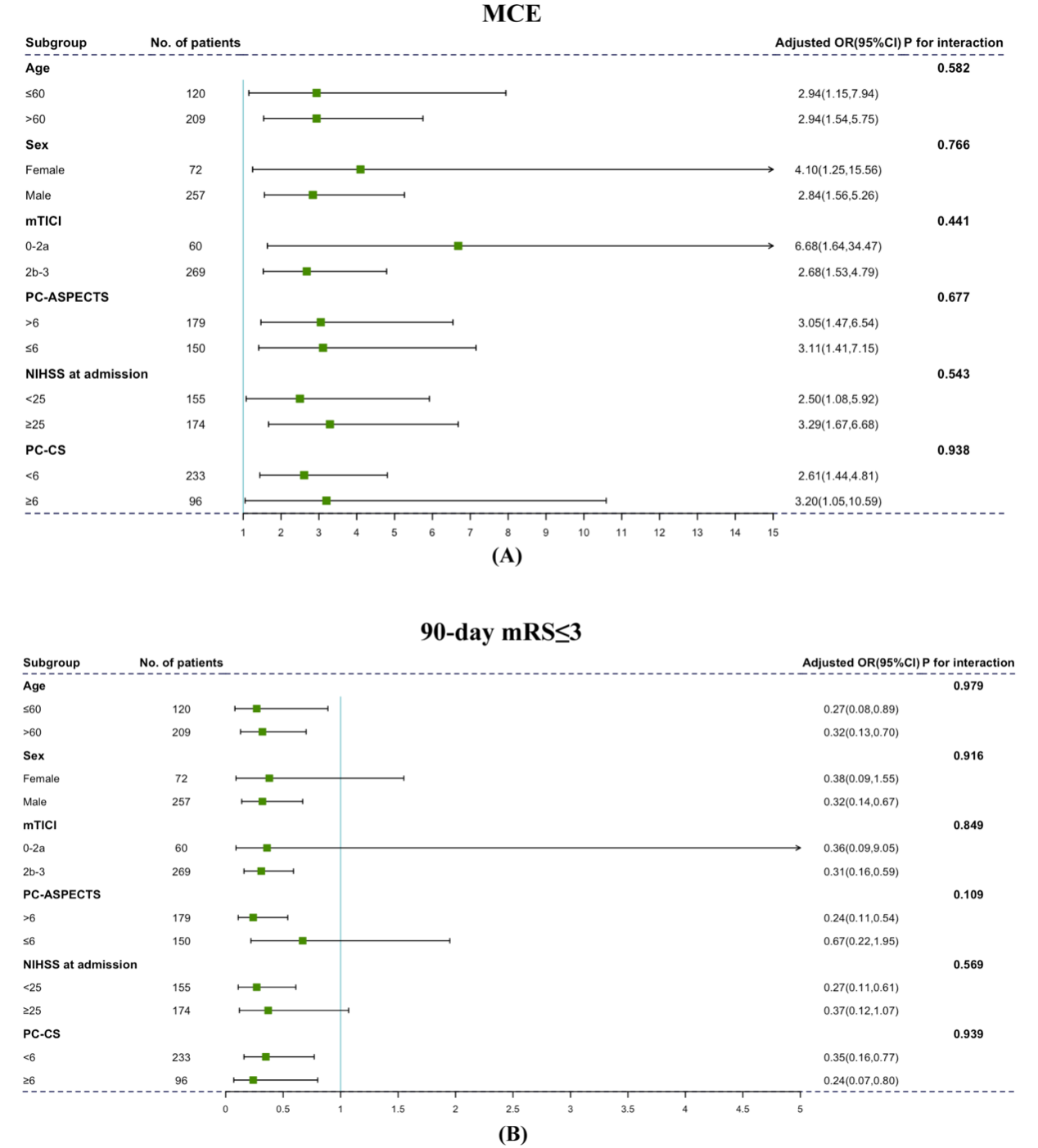


**Supplemental Figure 2.** Subgroup analysis of association between neutrophil count and malignant cerebellar edema (MCE, Supplemental Figure 2A) and favorable outcome (90-day mRS≤3, Supplemental Figure 2B).


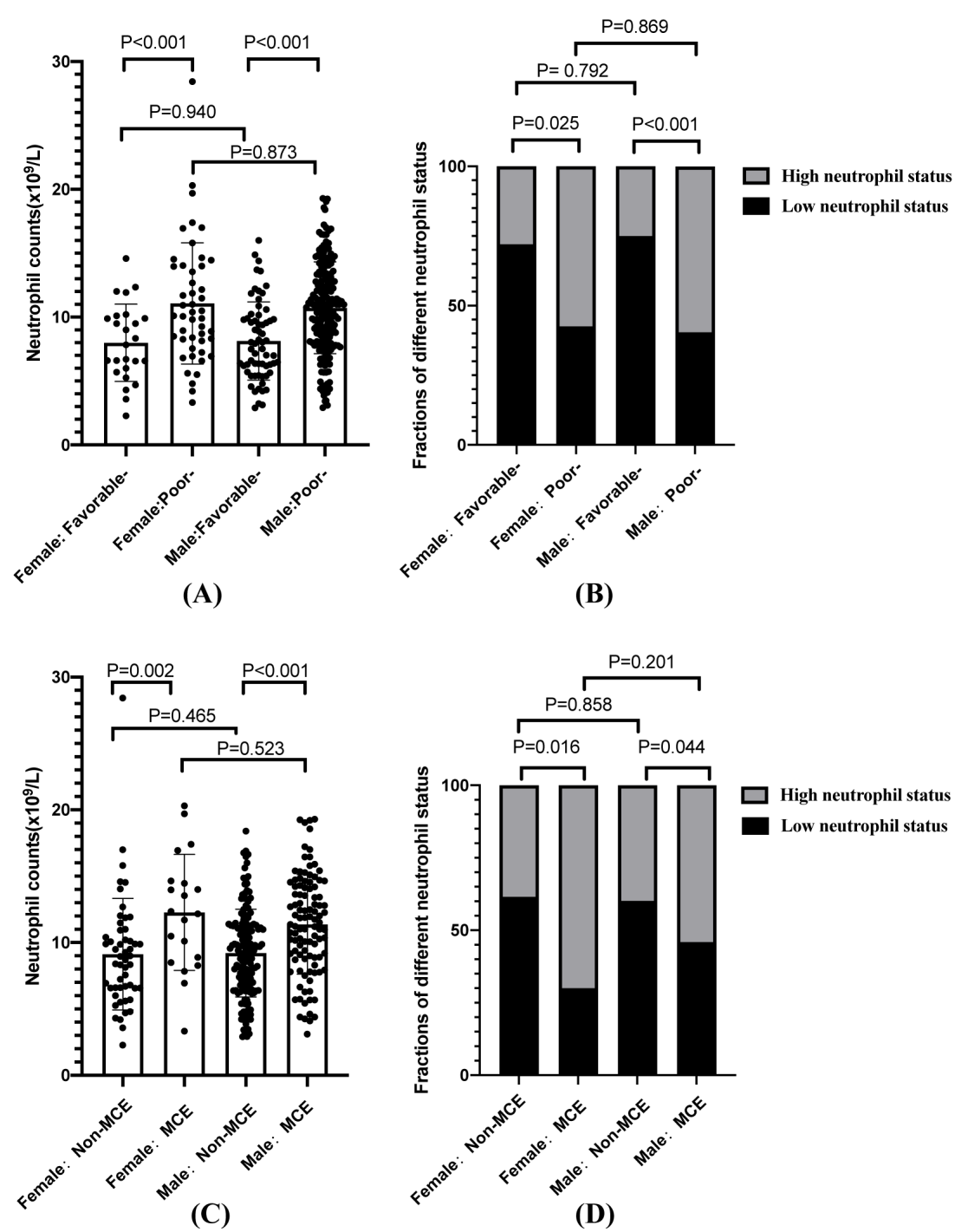


**Supplemental Figure 3.** Comparisons of prognostic values of neutrophil counts in 90-day outcome (A&B) and MCE (C&D) between different genders.
